# Long term deficiency of vitamin D in germ cell testicular cancer survivors

**DOI:** 10.18632/oncotarget.24925

**Published:** 2018-04-20

**Authors:** Lucia Nappi, Margaret Ottaviano, Pasquale Rescigno, Ladan Fazli, Martin E. Gleave, Vincenzo Damiano, Sabino De Placido, Giovannella Palmieri

**Affiliations:** ^1^ Department of Urologic Sciences, Vancouver Prostate Centre, University of British Columbia, Vancouver, BC, Canada; ^2^ Department of Medicine and Surgery, Division of Medical Oncology, Centro di Riferimento Tumori Rari Regione Campania, University of Naples “Federico II”, Napoli, Italy; ^3^ The Institute of Cancer Research, Prostate Targeted Therapy Group, Sutton, UK; ^4^ Department of Clinical Medicine and Surgery, University Federico II, Naples, Italy

**Keywords:** testicular cancer, vitamin D, testicular cancer survivors, long term side effects, quality of life

## Abstract

**Background:**

Cisplatin-based chemotherapy significantly improved the survival of patients with germ cell testicular cancer. However, long term side effects of chemotherapy have non-negligible impact on the quality of life of these young patients, who have a long life expectancy after being successfully treated.

**Materials and Methods:**

25-OH vitamin D, testosterone, FSH and LH of patients with testicular cancer were retrospectively evaluated and for each patient clinical information were collected. The tissue of 52 patients with germ cell tumors was analyzed for VDR expression by immunohistochemistry. The serum 25-OH vitamin D and VDR expression were correlated to the patients ‘clinical characteristics.

**Results:**

25-OH vitamin D was analyzed in 82 patients. Insufficient (< 30 ng/ml) levels were detected in 65%–85%, mild deficient (< 20 ng/ml) in 25%–36% and severe deficient (< 10 ng/ml) in 6%–18% of the patients over a median follow-up of 48 months. No difference in serum 25-OH vitamin D was detected over the follow-up time points. No correlation with histology, stage and type of treatment was found. The 25-OH vitamin D levels were not correlated to testosterone, FSH and LH levels. Interestingly, the expression of VDR was much higher in non seminoma than in seminoma tissue.

**Conclusions:**

Patients with testicular cancer have reduced vitamin D levels after the treatment of the primary cancer. Since long term hypovitaminosis D leads to high risk of fractures, infertility and cardiovascular diseases, we envision that vitamin D should be regularly checked in patients with testicular cancer and replaced if needed.

## INTRODUCTION

Germ cell testicular tumor (GCT) is the most frequent malignancy of young men and its incidence has significantly increased over the last 30 years [1–3]. GCTs are classified in seminoma and non seminoma (NS), both characterized by high sensitivity to cisplatin based chemotherapy which lead to a high cure rate (5 years overall survival above 95%) also for patients with advanced disease [4, 5]. Despite a long life expectancy, more concerns have been raised about the long term side effects related to chemotherapy and the quality of life of the survivor patients. Cardiovascular disease and second non-GCT malignancies are the most severe and potentially life-threating long term side effects of the GCTs treatments [6–8]. Other long-term side effects, including nephrotoxicity, neurotoxicity, hypogonadism, metabolic syndrome and reduced fertility are associated with high morbidity and their management and diagnosis have become part of the routine follow up [9]. However, many other abnormalities potentially affecting GCTs survivors’ quality of life, are either under-estimated or still unknown. Testicle is one of the most important extra-renal site for the activation of the vitamin D. CYP2R1, the 1-α-hydroxylase that converts the 25-OH vitamin D (25-OHvitD) into the 1,25-(OH)_2_ vitamin D, is highly expressed in the Leydig cells [10]. At the same time, testicle is a target-organ of vitamin D, as demonstrated by the high expression of vitamin D receptor (VDR) in the Sertoli cells [11]. In the testicle vitamin D regulates maturation and motility of the germ cells and controls fertility [12]. The activation of VDR by 1,25-(OH)_2_ vitamin D is essential for the regulation of calcium and phosphate metabolism and for the bone mineralization [13, 14]. In addition, vitamin D regulates several cell functions, including cell proliferation, differentiation and angiogenesis in prostate and other cancers [15–17]. In testicular cancer patients, low levels of 25-OHvitD have been correlated to low bone density and higher risk of fracture [18]. Moreover, hypovitaminosis D is related to low testosterone and infertility [19, 20]. Although few evidence demonstrated the presence of low levels of vitamin D in patients with GCT, [21] the correlation with the clinical features have not been described yet. In this study the timing of 25-OHvitD insufficiency/deficiency, the correlation with histology, stage, testosterone, gonadotropins levels and different treatments were analyzed in patients with GCTs.

## RESULTS

### Patients’ characteristics

Of the 137 patients reviewed, 82 had available serum 25-OHvitD measurements and were considered for the analysis. The patients’ characteristics are summarized in Table 1. All the seminoma patients had primary testis tumor, while 3 of the NS had extra-gonadal GCT. Five patients had bilateral testicular cancer. The median age of the population was significantly higher in the seminoma cohort compared to the NS patients (*p* < 0.001). Majority of patients had clinical stage I (CSI) disease. Metastatic GCT was more frequent among NS than in the seminoma patients (stage II: 7.3% seminoma, 20.7% NS; stage III: 3.6% seminoma, 16.9% NS). Most of the CSI patients received adjuvant treatment: carboplatin (*N* = 15) or radiation therapy (*N* = 3) for seminoma and 2 cycles of BEP (Bleomycin, Etoposide, Cisplatin) (*N* = 16) for NS. Eleven patients with CSI were managed with active surveillance. The patients with metastatic disease were treated with 3 or 4 cycles of BEP chemotherapy. Of notice, 1 patient with stage III NS received 2 cycles of BEP because of toxicity. The median follow-up was 48 months (3–168 months, IQR: 16.5–96 months).

**Table 1:** Patients’ characteristics

	Seminoma		Non seminoma	
	*N*	%	*N*	%
**Histology**	33	40.2	49	59.7
**Primary tumor site**				
Testis	33	40.2	46	56
- bilateral	3	3.6	2	2.4
Mediastinum	-	-	2	2.4
Other	-	-	1	1.2
**Age, years**				
Median	35		27.5	
Range	22–57		17–42	
IQ1–3	30–39		23–34	
**Stage**				
I	26	31.7	19	23.1
II	6	7.3	17	20.7
III	3	3.6	14	16.9
**Treatment**				
Surveillance	8	9.7	3	3.6
Radiation (CSI)	3	3.6	0	0
carboplatin 1 cycle	15	18.2	0	0
BEP x 2	1^a^	1.2	16	19.5
BEP x 3	7	8.5	18	21.9
BEP x 4	1	1.2	12	14.6
**Follow-up, months**				
Median	51		42	
Range	3–168		3–132	
IQ1–3	17.25–111		15–84	

^a^The patient had metastatic seminoma but received 2 cycles of BEP because of toxicity.

### Patients with GCTs have long term low serum 25-OHvitD levels

**Figure 1:**
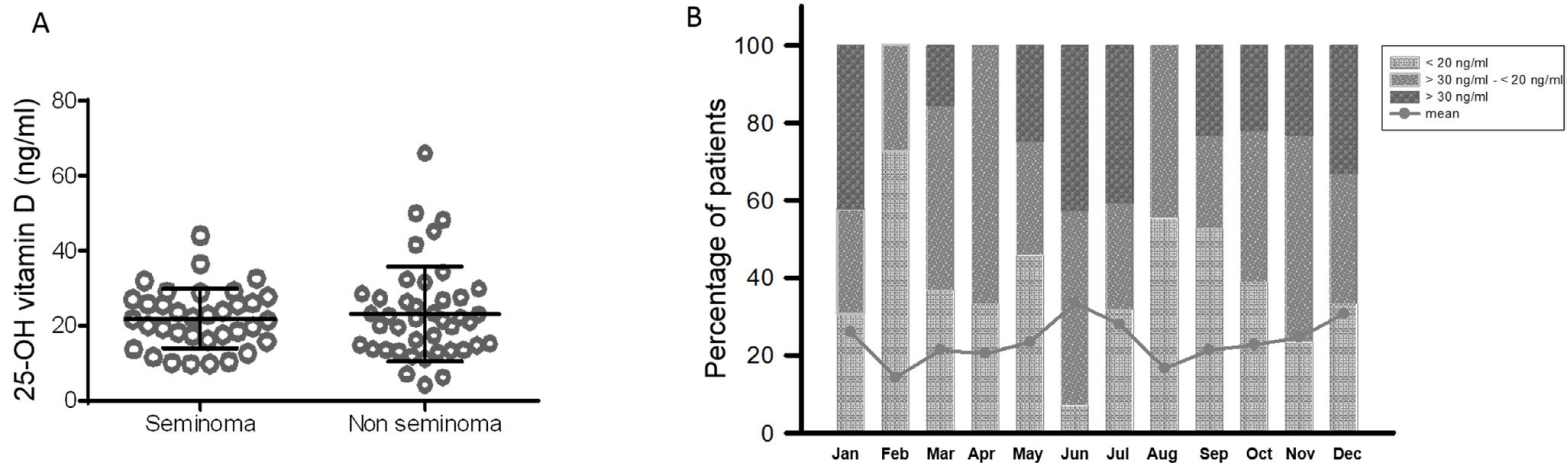
Patients with testicular cancer have seasonal unchanged low serum levels of 25-OH vitamin D (**A**) The serum levels of 25-OH vitamin D were evaluated in patients with Germ Cell Tumors. No differences were observed between seminoma and non seminoma patients (*p* = 0.9980, Mann-Whitney test). Each sphere represents a measurement. Horizontal bar: medium; vertical bars: standard deviation. (**B**) The distribution of the serum levels of 25-OH vitamin D measurements was analyzed for each month of the year. The percentage of sufficient (≥ 30 ng/ml), insufficient (> 30 ng/ml – ≤ 20 ng/ml) and deficient (< 20 ng/ml) values was evaluated according to the month of blood withdrawal. The linear graph shows the change of the medium 25-OH vitamin D measurements for each month of the year.

**Figure 2:**
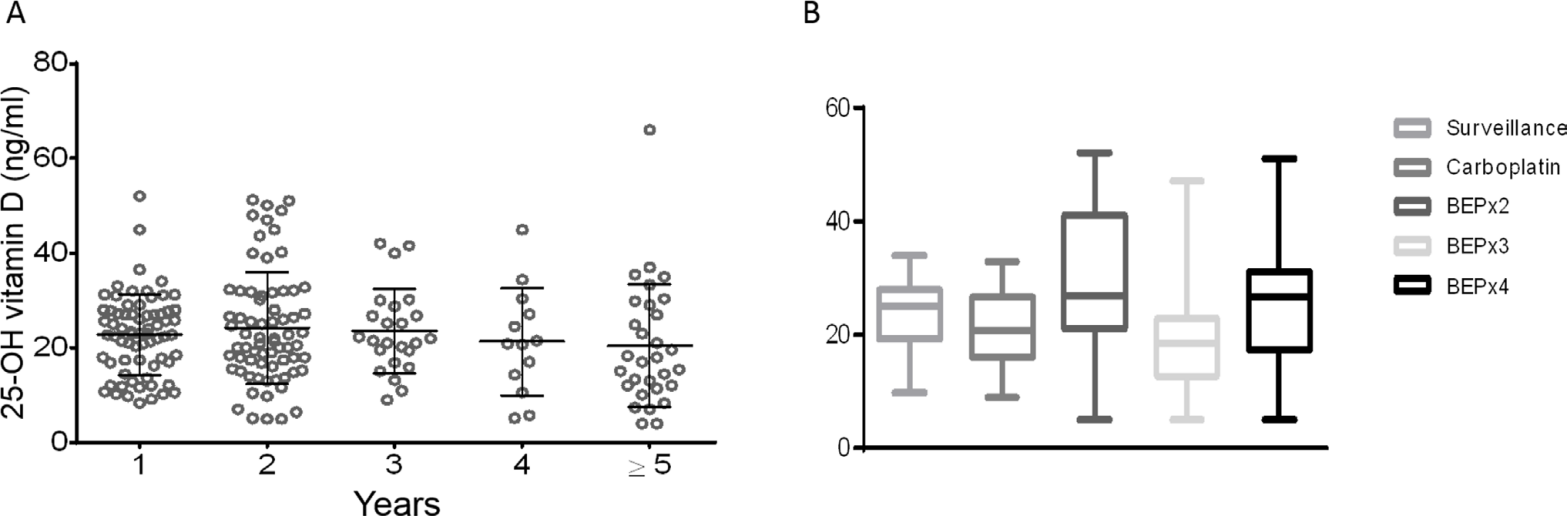
Serum levels of 25-OH vitamin D are stably low over ≥ 5 years from the diagnosis of testicular cancer (**A**) The serum levels of 25-OH vitamin D collected at different follow-up points (1- ≥ 5 years) from the primary diagnosis of testicular cancer. No differences were observed over the follow-up time points (*p* = 0.55, one way ANOVA with Tukey's multiple comparisons test). Each sphere represents a measurement. Horizontal bar: medium; vertical bars: standard deviation. (**B**) The serum levels of 25-OH vitamin D were correlated to surveillance, chemotherapy with carboplatin, and BEP chemotherapy (2, 3 or 4 cycles). No statistically difference was observed between the patients managed with surveillance or treated with chemotherapy (*p* = 0.96, one way ANOVA with Bonferroni correction for the multiple comparisons test). Horizontal bar: medium; vertical bars: standard deviation.

The patients had a median of three 25-OHvitD measurements (range 1–5) with 58% of patients having ≥ 3 available values (Supplementary Table 1). The medium value of 25-OHvitD was 22.7 ng/ml and no differences were observed between seminomas and NS patients (*p* = 0.998, Supplementary Table 2 and Figure 1A). The exposition to the UV did not affect the medium serum levels of 25-OHvitD. The levels were in fact not significantly different when the measurement was done during the low or high season (respectively, 22.4 ng/ml vs 23.4 ng/ml, *p* = 0.63 for seminoma and 24.4 ng/ml vs 25.1 ng/ml, *p* = 0.83 for NS) (Supplementary Table 3). Moreover, to further study the effect of the UV exposition on vitamin D serum fluctuation, we analyzed the 25-OHvitD levels for each month. Contrarily to what reported in the general population, [22] the 25-OHvitD serum levels were only marginally affected by the seasons, with the average values below 30 ng/ml also during summer months (Figure 1B). The levels of 25-OHvitD were then analyzed at different follow-up time points. The majority of patients had 25-OHvitD evaluated during the first 2 years (57% and 53% of patients evaluated at year 1 and 2, respectively), 24% and 13% at year 3 and 4. Interestingly, 31.7% had 25-OHvitD measured after ≥ 5 years from the first diagnosis of the GCT. Insufficient (< 30 ng/ml) levels of 25-OHvitD were detected in 65%–85%, of the patients at the follow-up time points analyzed. Of notice, a significant percentage of the patients showed a mild deficiency (< 20 ng/ml) (25%–36%) while 6%–18% of the patients presented a severe (< 10 ng/ml) deficiency (Table 2). Interestingly, no differences were observed over the years of follow up (*p* = 0.5533, Figure 2A) and between seminomas and NS patients (*p* = 0.7102 for seminoma and *p* = 0.4023 for NS, Supplementary Figure 1A, 1B). Of notice, 80% of the patients with the longest follow up (60–168 months) presented levels of 25-OHvitD < 30 ng/ml, with 50% and 15% of them having mild and severe deficiency, respectively.

**Table 2:** Percentage of the patients with insufficient/deficient 25-OH vitamin D levels

	Year 1	Year 2	Year 3	Year 4	Year ≥ 5
**Patients % (N)**	57.3 (47)	53.6 (44)	24.3 (20)	13.4 (11)	31.7 (26)
**Patients with 25-OH vitamin D < 30 ng/ml %**	85.1	65.9	80	72.7	80.7
**- Patients with 25-OH vitamin D < 10 ng/ml**	6.3	11.3	5	18.1	15.3
**- Patients with 25-OH vitamin D < 20 - ≥ 10 ng/ml**	36.1	31.8	25	27.2	34.6
**- Patients with 25-OH vitamin D < 30 - ≥ 20 ng/ml**	42.5	22.7	50	27.2	30.7
**Patients with 25-OH vitamin D ≥ 30 ng/ml %**	14.8	34	20	27.2	19.2

### Vitamin D deficiency is not correlated to testosterone and FSH-LH levels

Testosterone, FSH and LH levels were evaluated at the same time points of vitamin D assessment and are showed in Supplementary Figure 2. The medium levels of total testosterone was 5.19 ng/ml (5.49 ng/ml and 4.99 ng/ml, for seminoma and NS, respectively). Most of the patients analyzed had normal levels of testosterone and only 10% and 8% during the first 2 years of follow-up had testosterone levels below the normal range. While the medium LH values were within the normal range (medium 8.54 mIU/ml) the FSH medium levels were higher than the normal values (medium 20.66 mIU/ml). A significant percentage of patients (31%–65%) presented stably high levels of FSH over the follow-up time points. No statically significant correlation between 25-OHvitD levels and testosterone, FSH and LH during the follow-up points analyzed was found in both seminoma and NS patients (Supplementary Tables 4–5).

### 25-OHvitD serum levels are not affected by the tumor specific treatments

Usually the onset of the adverse effects varies according to treatment type and intensity [9]. To understand if the treatment received influenced the serum levels of 25-OHvitD, its levels were correlated to the treatment option (surveillance or chemotherapy). No differences were described among the patients treated with surveillance or chemotherapy over 5 or more years of follow-up (*p* = 0.96, Figure 2B).

### Serum 25-OHvitD levels do not correlate to the stage of disease

The serum levels of 25-OHvitD were then correlated to the clinical stage of patients with GCTs. The levels of 25-OHvitD of both seminoma and NS patients at the clinical stage I were compared to the 25-OHvitD levels of the patients with metastatic disease (stage II-III). No statistically significant difference was demonstrated (*p* = 0.5289, Supplementary Figure 3).

### VDR is differentially expressed in GCTs

**Figure 3:**
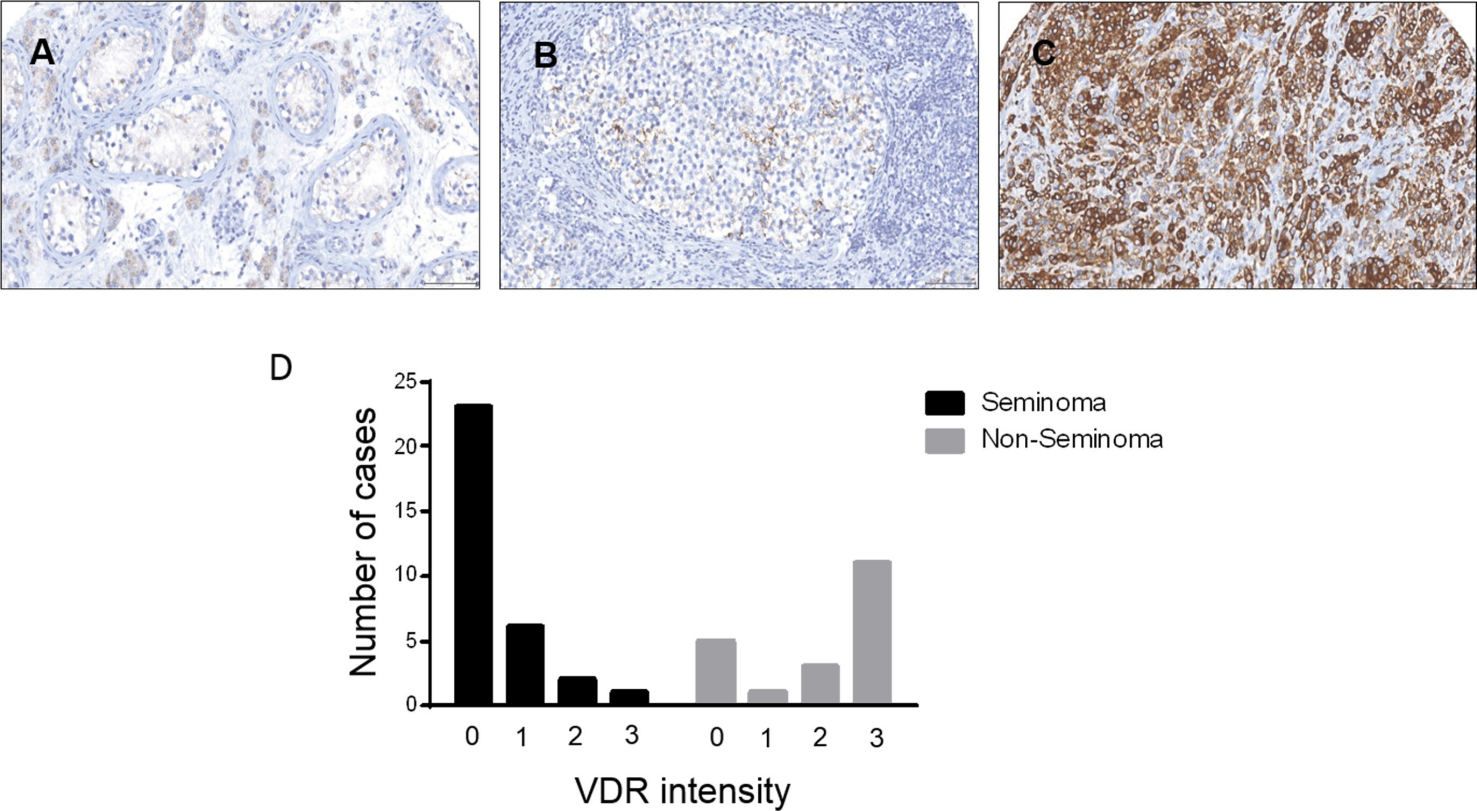
Vitamin D receptor expression in testicular germ cell tumors Immuno-expression of VDR in (**A**), Benign non tumoral testis with weakly reactive leydig cells. (**B**), shows Seminoma and VDR expression in some tumor cells and (**C**), Shows strong immunoreactivity of tumor cells in Embryonal Carcinoma. (**D**) VDR expression was significantly higher in Non seminoma patients compared to seminoma (*p* < 0.0001, χ^2^ test).

To assess if the expression of VDR was correlated to any clinical features of GCTs, its expression was analyzed by IHC in 52 patients with GCTs, 31 seminoma and 21 NS. Benign tissue was available for 6 of the specimen and was used as control. As previously reported, the expression of VDR was higher in germ tumor cells compared to the normal germ cells (Figure 3A, 3B). Interestingly, the expression of VDR was significantly higher in NS than in seminoma (*p* < 0.0001, Figure 3C, 3D). No difference in VDR expression was observed between CSI and metastatic patients in both seminoma and NS patients (*p* = 0.2273 and 0.5622, respectively) and between the patients who did or did not have a relapse of the disease (*p* = 0.6586, Supplementary Figure 4).

## DISCUSSION

Although there is no consensus on optimal serum levels of 25-OHvitD, a concentration < 30 ng/ml and < 20 ng/ml is respectively considered insufficient and deficient by most of the endocrinology societies [23, 24]. Few case-control studies reported low levels of vitamin D in patients with GCT compared to healthy matching controls [18, 21]. We confirmed that reduced vitamin D levels are frequently observed among patients with GCTs, with 65%–85% presenting levels < 30 ng/ml, 25%–36% < 20 ng/ml and 6%–18% < 10 ng/ml. We also defined the timing of vitamin D serum levels reduction. In our cohort 25-OHvitD values < 30 ng/ml were observed in 85% of the patients during the first year and remained stably low during the follow up, with 50% of the patients still presenting low levels after ≥ 5 years from the diagnosis. In healthy people the exposition to the UV radiation increases the levels of vitamin D [22, 25]. Interestingly, in GCTs patients, the vitamin D deprivation status was not affected by the UV exposition, as demonstrated by stable low levels of vitamin D during the high season months (May-October). Hypogonadism is one of the well-established long term sequela of GCT [26] especially in patients treated with chemotherapy [27]. Several studies reported the association of low 25-OHvitD levels and hypogonadism [19, 20, 28]. However, we did not observe any correlation between vitamin D and testosterone, LH and FSH. Moreover, while testosterone and LH levels were within the normal range in most of the patients, FSH levels were beyond normal range in a significant percentage of them. Elevated FSH levels have been described in testicular cancer survivors after orchiectomy alone and especially after radiation/chemotherapy and have been correlated to reduced fertility [29]. VDR expression is controlled by FSH in Sertoli cells where contributes to germ cells maturation and motility [10, 11]. In a condition of stably low serum levels vitamin D, the increase of FSH may represent a positive feedback to increase VDR expression and promote the spermatogenesis. While most part of the known long term side effects are related to the type and the intensity of the treatment, a difference in 25-OHvitD levels was not observed in the patients managed with surveillance, treated with single agent carboplatin or poly-chemotherapy. This data suggests that, unlike the other long term side effects, the hypovitaminosis D is not drug-related and thus possibly a consequence of the orchiectomy. However, a pre-orchiectomy deficiency cannot be excluded. Stable long-term insufficient/deficient vitamin D levels may contribute to the impairment of quality of life and morbidity in a population with a long life expectancy such as GCT patients. Low levels of 25-OHvitD are correlated to low bone densitometry, [18, 30] high risk of vertebral fractures [31], to an increased infertility [32, 33] and cardiovascular risk [34, 35]. In addition, low levels of 25-OHvitD correlate to a bad prognosis in the afro-american patients with testicular and prostate cancer [36]. Although no correlation was observed between VDR expression and 25-OHvitD serum levels and extension of the disease, a higher VDR expression was observed in NS compared to the seminoma patients, suggesting a pre-orchiectomy vitamin D metabolism abnormality and/or a possible role in the pathogenesis of GCTs.

Despite some limitations (retrospective collection of the data, small number of patients, lack of pre-orchiectomy 25-OHvitD serum levels, absence of a healthy control groups), our study confirms that patients with GCT have reduced serum levels of 25-OHvitD, with a non-negligible percentage of these patients presenting mild/severe deficiency. We defined, for the first time, the timing of vitamin D insufficiency/deficiency and the absence of correlation between low 25-OHvitD levels and the treatment received, clinical stage, histology, testosterone, gonadotropins levels. Considering that GCT patients have a high rate of cure and a long expectancy of life, we envision that vitamin D should be checked regularly in patients with testicular cancer and therapeutically corrected to reduce the risk of long-term effects correlated to low levels of vitamin D. Further studies with evaluating pre-orchiectomy 25-OHvitD in a larger sample size and including a healthy control group would be helpful to confirm and further clarify the etiology of this hypovitaminosis.

## MATERIALS AND METHODS

### Population

The serology data of 137 patients with classic seminoma or NS (embryonal carcinoma, yolk sac tumor, choriocarcinoma or mixed seminoma and NS), treated at the Rare Tumor Reference Centre of Campania Region of the University of Naples “Federico II”, from January 2006 to January 2013, were retrospectively reviewed. Patients with at least one 25-OHvitD measurement and a minimum follow up of 3 months were included. Post-orchiectomy serum levels of 25-OHvitD were evaluated at: 3, 6, 9, 12 months (year 1); 15, 18, 24 months (year 2); 30, 36 months (year 3); 40, 48 months (year 4); 60–180 months (year ≥ 5). To analyze the fluctuation of 25-OHvitD related to the UV radiation exposition, the values of 25-OHvitD were divided in high season (from May to October) and low season (from November to April) [37]. The patients were defined to have sufficient (≥ 30 ng/ml), insufficient (< 30 – ≥ 20 ng/ml), mildly deficient (< 20 ng/ml – ≥ 10 ng/ml) or severe deficient (< 10 ng/ml) 25-OHvitD according to the Endocrine Society guidelines and Institute of Medicine's standards [23, 38]. Testosterone, FSH and LH were also evaluated (normal values: 2.4–12 ng/ml, 1.3–19.3 mIU/ml and 1.8–12 mIU/ml, respectively). A signed consent form was collected from each patient, according to the ethic committee regulation of the University “Federico II” of Naples (protocol 150/14).

### Human tissue samples

Fifty-two cases of GCTs with follow-up data diagnosed between 2003 and 2010 were identified from the archives of Vancouver General Hospital, Vancouver, British Columbia. The paraffin-embedded tissue blocks were used to construct a duplicate core Tissue microarray (TMA).

### Immunohistochemistry

Immunohistochemistry (IHC) staining was conducted by Ventana autostainer model Discover XT (Ventana Medical System) with enzyme-labeled biotin streptavidin system and solvent-resistant DAB Map kit by using rabbit polyclonal VDR (H-81; sc-9164) antibody from Santa Cruz Biotechnology, Inc.

### Digital imaging and scoring

All stained slides were digitalized with the SL801 autoloader and Leica SCN400 scanning system (Leica Microsystems; Concord, Ontario, Canada) at magnification equivalent to ×40. The images were subsequently stored in the SlidePath digital imaging hub (DIH; Leica Microsystems) of the Vancouver Prostate Centre. The scoring method used was based on assigning a value on a four-point scale to each immunostain. Descriptively, 0 represents no staining by any tumor cells (negative), 1 represents a faint or focal, questionably present stain (weak), 2 represents a stain of convincing intensity in a minority of cells (moderate), and 3 a stain of convincing intensity in a majority of cells (strong).

### Statistical analysis

One way ANOVA with Tukey's multiple comparisons test or Bonferroni correction was used to analyze the 25-OHvitD serum levels of the GCTs patients and their correlation with timing and clinic-pathological characteristics. Nominal characteristics were compared using Mann-Whitney test. The correlation between serum 25-OHvitD and testosterone, FSH and LH was analyzed using Spearman test. Chi square test was used for correlation of IHC score and clinical features. The data was analyzed using Prisma 6 software.

## SUPPLEMENTARY MATERIALS FIGURES AND TABLES



## Abbreviations

GCT: Germ Cells testicular Tumor
NS: Non Seminoma
CYP2R1: Cytochrome P450 Family 2 Subfamily R Member 1
25-OHvitD: 25-OH vitamin D
VDR: Vitamin D Receptor
CSI: Clinical Stage I
BEP: Bleomycin, Etoposide, Cisplatin
UV: Ultraviolet
FSH: Follicle-stimulating hormone
LH: Luteinizing hormone
IHC: Immunohistochemistry
TMA: Tissue Micro Array.
