# Supplementary material for: Long term deficiency of vitamin D in germ cell testicular cancer survivors
Source: Oncotarget. 2018 Apr 20;9(30):21078–85. doi: 10.18632/oncotarget.24925 (PMC5940414; doi:10.18632/oncotarget.24925)
Supplement: Supplementary file 1 [file oncotarget-09-21078-s001.pdf]

Long term deficiency of vitamin D in germ cell testicular cancer survivors

SUPPLEMENTARY MATERIALS

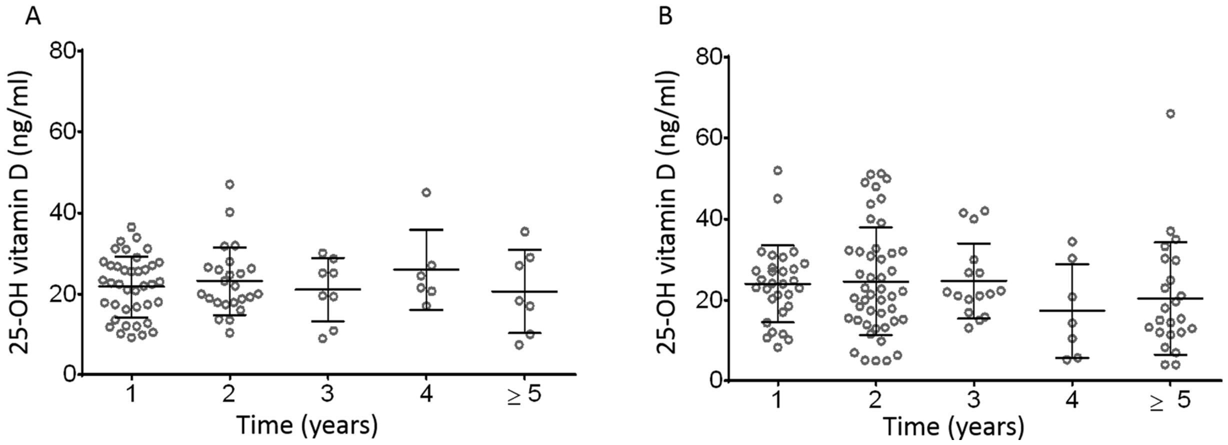

Supplementary Figure 1: Serum levels of 25-OH vitamin D are stably low over ≥ 5 years from the diagnosis in seminoma and non seminoma testicular cancer patients. The serum levels of 25-OH vitamin D collected after 1- ≥ 5 years from the diagnosis of testicular cancer were compared. No differences were observed at 1, 2, 3, 4 or ≥ 5 years in both (**A**) seminoma (p = 0.71) and (**B**) non seminoma (p = 0.40) patients. One way ANOVA with Tukey's multiple comparisons test. Each sphere represents a measurement. Horizontal bar: medium; vertical bars: standard deviation.

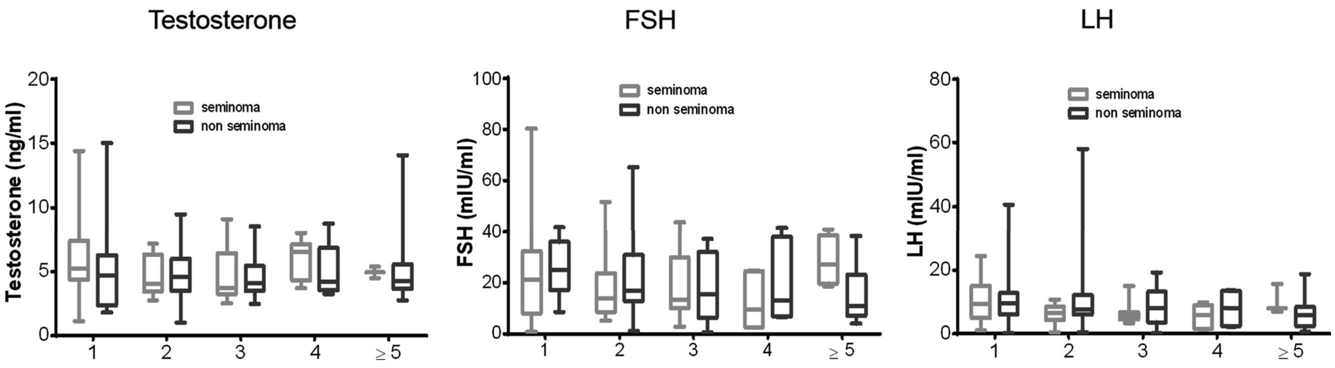

Supplementary Figure 2: Serum levels of testosterone, FSH and LH in the testicular cancer patients survivors. The serum levels of testosterone, FSH and LH were collected at different time points (from 1 to ≥ 5 years) after the orchiectomy. Horizontal bar: medium; vertical bars: standard deviation.

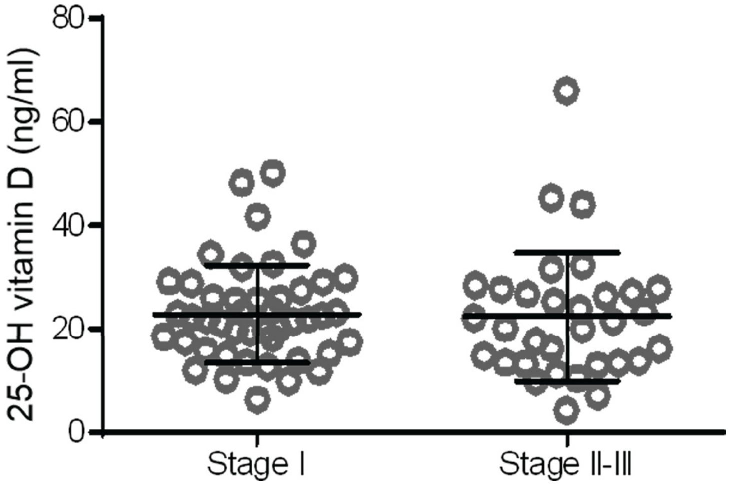

Supplementary Figure 3: Serum levels of 25-OH vitamin D are not correlated to chemotherapy and stage of the disease. The serum levels of 25-OH vitamin D were evaluated in patients with localized stage I or metastatic (stage II–III) Germ Cell Tumors. No differences were observed between the two groups (p = 0.5289, Mann-Whitney test). Each sphere represents a measurement. Horizontal black bar: medium; vertical bars: standard deviation.

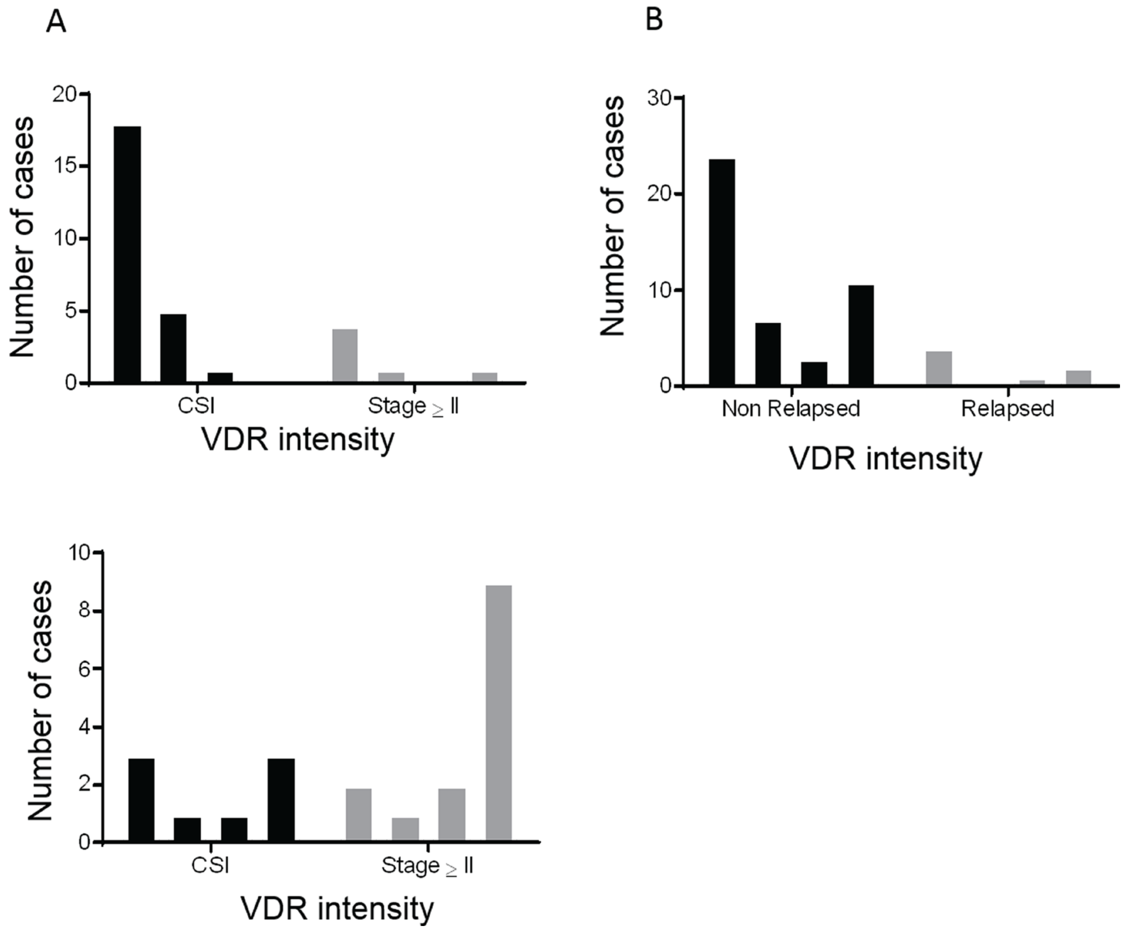

Supplementary Figure 4: VDR expression is not correlated to stage of the disease or risk of relapse in testicular Germ Cell tumors. (**A**) Immunoexpression of VDR in patient with stage I or metastatic disease was compared in seminoma and non seminoma patients. No statistically significant difference was observed in seminoma (upper panel, χ^2 test, p = 0.22) and non seminoma patients (lower panel, χ^2 test, p = 0.56). (**B**) VDR expression was analyzed in patients who presented or did not present a relapse of the disease after the primary treatment for GCT. No difference was observed (χ^2 test, p = 0.65).

Supplementary Table 1: 25-OH vitamin D serum measurements

Number of 25-OH vitamin D measurements	Number of patients	Percentage of patients
1	21	25.6
2	13	15.8
3	38	46.3
4	9	10.9
5	1	1.2

Supplementary Table 2: 25-OH vitamin D serum concentration

	Medium	SD
25-OH vitamin D (ng/ml)	22.7	10.6
- Seminoma	21.9	7.9
- Non Seminoma	23.4	12.5

Supplementary Table 3: Seasonal 25-OH vitamin D levels

	Seminoma	Non Seminoma	
	Medium (SD)	Medium (SD)	***T*-test**
High season (May-October)	22.4 (8.8)	24.4 (12.9)	*p* 0.4
Low season (November-April)	23.4 (7.8)	25.1 (14)	*p* 0.6

Supplementary Table 4: Testosterone, LH and FSH serum levels

	ALL	SEMINOMA	NON SEMINOMA
	Medium (SD)	Medium (SD)	Medium (SD)
Testosterone (ng/ml)	5.1 (2.5)	5.4 (2.5)	4.9 (2.4)
LH (mIU/ml)	8.5 (7.2)	7.2 (4.9)	9.3 (8.2)
FSH (mIU/ml)	20.6 (13.3)	20.8 (14.5)	20.5 (12.7)

Supplementary Table 5: Correlation between 25-OH vitamin D and testosterone, FSH and LH

	Year 1		Year 2		Year 3		Year 4		Year ≥ 5	
	r	*p*	*r*	*p*	*r*	*p*	*r*	*p*	*r*	*p*
25-OHvitD vs. testosterone	0.1579	0.2735	−0.3173	0.1728	0.1291	0.5985	−0.1259	0.6999	−0.1018	0.6782
25-OHvitD vs. FSH	0.01511	0.9120	0.3420	0.1102	0.09830	0.7264	−0.05455	0.8812	0.1353	0.5481
25-OHvitD vs. LH	−0.06437	0.6406	−0.01073	0.9622	−0.2395	0.3869	−0.4909	0.1294	0.1966	0.3805
